# UBE2O promotes hepatocellular carcinoma cell proliferation and invasion by regulating the AMPKα2/mTOR pathway

**DOI:** 10.7150/ijms.63220

**Published:** 2021-10-11

**Authors:** Zhan Shi, Runkun Liu, Qiliang Lu, Zhi Zeng, Yang Liu, Junjun Zhao, Xin Liu, Lijie Li, Hui Huang, Yingmin Yao, Dongsheng Huang, Qiuran Xu

**Affiliations:** 1 The Second Clinical Medical College, Zhejiang Chinese Medical University, Hangzhou 310053, China.; 2 The Key Laboratory of Tumor Molecular Diagnosis and Individualized Medicine of Zhejiang Province, Zhejiang Provincial People's Hospital, Affiliated People's Hospital, Hangzhou Medical College, Hangzhou 310014, China.; 3 Department of Hepatobiliary Surgery, The First Affiliated Hospital of Xi'an Jiaotong University, Xi'an 710061, China.; 4 The Medical College of Qingdao University, Qingdao, 266071, China.; 5 Graduate Department, Bengbu Medical College, Bengbu 233030, China.; 6 Department of Obstetrics and Gynaecology, Affiliated Zhejiang Hospital, Zhejiang University School of Medicine, Hangzhou 310013, China.; 7 Affiliated Quzhou People's Hospital, Zhejiang Chinese Medical University, Quzhou 324002, China.

**Keywords:** Hepatocellular carcinoma, UBE2O, AMPKα2, mTOR pathway, Tumor progression

## Abstract

The ubiquitin-conjugating enzyme (E2) is a critical component of the ubiquitin-proteasome system and regulates hepatocarcinogenesis by controlling protein degradation. Ubiquitin-conjugating enzyme E2 O (UBE2O), a member of the E2 family, functions as an oncogene in human cancers. Nevertheless, the role of UBE2O in hepatocellular carcinoma (HCC) remains unknown yet. Here, we demonstrated that the UBE2O level was markedly upregulated in HCC compared with adjacent noncancerous tissues. UBE2O overexpression was also confirmed in HCC cell lines. UBE2O overexpression was prominently associated with advanced tumor stage, high tumor grade, venous infiltration, and reduced HCC patients' survivals. UBE2O knockdown inhibited the migration, invasion, and proliferation of HCCLM3 cells. UBE2O overexpression enhanced the proliferation and mobility of Huh7 cells. Mechanistically, UBE2O mediated the ubiquitination and degradation of AMP-activated protein kinase α2 (AMPKα2) in HCC cells. UBE2O silencing prominently increased AMPKα2 level and reduced phosphorylated mechanistic target of rapamycin kinase (p-mTOR), MYC, Cyclin D1, HIF1α, and SREBP1 levels in HCCLM3 cells. UBE2O depletion markedly activated the AMPKα2/mTOR pathway in Huh7 cells. Moreover, AMPKα2 silencing reversed UBE2O downregulation-induced mTOR pathway inactivation. Rapamycin, an inhibitor of mTOR, remarkably abolished UBE2O-induced mTOR phosphorylation and HCC cell proliferation and mobility. To conclude, UBE2O was highly expressed in HCC and its overexpression conferred to the poor clinical outcomes of patients. UBE2O contributed to the malignant behaviors of HCC cells, including cell proliferation, migration, and invasion, by reducing AMPKα2 stability and activating the mTOR pathway.

## Introduction

The most recent IARC data shows that liver cancer is the fifth commonest cancer and the 2^nd^ leading cause of cancer-related deaths in China 1. About 90% of liver cancer is hepatocellular carcinoma (HCC), which has a poor prognosis with a five-year survival of less than 20% after surgery in China 2. To improve the clinical outcomes of patients with HCC, researchers focus on the investigation of HCC pathogenesis and the discovery of new targeted drugs for HCC.

Ubiquitination is an important post-translational modification of protein and plays an essential role in controlling substrate degradation 3. The ubiquitin proteasome system (UPS) contains ubiquitin-activating enzyme (E1), ubiquitin-conjugating enzyme (E2), ubiquitin ligase (E3), proteasome and deubiquitinating enzymes (DUBs) 4. E1 activates ubiquitin (Ub) using ATP and transfers it to E2, E2 interacts with a specific E3 and transfers Ub to the target protein, and finally the ubiquitinated substrate is degraded by 26S proteasome 5. Moreover, DUBs remove Ub from substrate proteins to reverse the function of Ub ligases 6. Increasing researches demonstrate the critical functions of ubiquitin modifying enzymes in the occurrence and progression of human HCC 7-9. For instance, the E3-ubiquitin ligase tumor necrosis factor receptor-associated factor 7 (TRAF7) is highly expressed in HCC, and its overexpression predicts the poor prognosis of HCC 10. TRAF7 directly targets Krüppel-like factor 4 (KLF4) for ubiquitin proteolysis to facilitate HCC progression 10. F-box and WD repeat domain containing 7 (FBXW7) is identified as a tumor-suppressive factor and exerts an inhibitory role in HCC cell growth by regulating Yes-associated protein (YAP) degradation 8. The upregulation of ubiquitin-conjugating enzyme E2 S (UBE2S) and its association with poor prognosis are reported by a previous study 11. UBE2S contributes to HCC progression by interacting with tripartite motif-containing 28 (TRIM28) and subsequently promoting the ubiquitination of p27 12. UBE2L3 functions as an oncogene and contributes to the proliferation and apoptosis resistance of tumor cells by suppressing the glycogen synthase kinase 3 beta (GSK3β)/p65 pathway in HCC 13.

Recent studies report the oncogenic or tumor-suppressive role of UBE2O in human cancers 14-19. For example, UBE2O mediates the multi-monoubiquitination of BRCA1-associated protein 1 (BAP1) and subsequently reduces its cytoplasmic retention 15. UBE2O participates in mixed-lineage leukemia (MLL) progression by regulating MML protein degradation 14. UBE2O targets c-Maf for ubiquitination and degradation to repress the tumor growth of myeloma 17. Mxi1 is identified as a substrate of UBE2O and mediates the tumor-promoting role of UBE2O in lung cancer 19. AMP-activated protein kinase α2 (AMPKα2) is a classical target protein of UBE2O in skeletal muscle and human cancers 16, 18, 20. UBE2O activates the mechanistic target of rapamycin kinase (mTOR) signaling pathway by ubiquitinating and destabilizing AMPKα2 in human cancers 16, 18. Nevertheless, the role of UBE2O in HCC and the underlying mechanism are unclear yet.

Here, we determined UBE2O expression in HCC and adjacent nontumor tissues and analyzed its prognostic significance. We investigated the effects of UBE2O on cell migration, invasion, and proliferation. The AMPKα2/mTOR pathway regulated by UBE2O was confirmed in HCC cells. Our data showed that the level of UBE2O was reduced in HCC specimens and associated with a poor prognosis. UBE2O enhanced the malignant behaviors of tumor cells by regulating the AMPKα2/mTOR pathway in HCC.

## Material and methods

### HCC patients and specimens

We collected eighty pairs of tumors and adjacent nontumor tissues from HCC patients who underwent surgical resection at the 1^st^ Affiliated Hospital of Xi'an Jiaotong University after obtaining written informed consent. All patients were not treated with pre-operative treatment and had complete clinical information, including follow-up data. The pathologists confirmed all specimens, and we subsequently maintained them in -80 °C for RT-qPCR analysis and fixed them with 10% formalin for immunohistochemistry. The Ethics Committee of the 1^st^ Affiliated Hospital of Xi'an Jiaotong University approved the current study protocols. Table 1 contains HCC patients' clinical characteristics.

### Cell culture and plasmid transfection

We previously preserved five human HCC cell lines (HCCLM3, Hep3B, HepG2, MHCC97H, and Huh7) in our lab 21. The normal hepatic cell line MIHA was obtained from bnbio (Beijing, China). All cell lines were cultured in DMEM medium (Gibco, Gaithersburg, MD, USA) supplemented with 10% fetal bovine serum (FBS, Gibco), streptomycin (100 mg/mL), and penicillin (100 U/mL) in a 5% CO_2_ incubator at 37 °C. We generated UBE2O expression plasmid by inserting cDNA into the pcDNA3.1 vector (Invitrogen, Carlsbad, CA, USA). UBE2O shRNAs (sh-UBE2O#1 and sh-UBE2O#2), AMPKα2 shRNA (sh-AMPKα2), and nontargeting shRNA (sh-NC) were provided by GenePharma (Shanghai, China). The vectors were transfected into HCC cells using Qiagen Effectene transfection reagent (Valencia, CA, USA). The shRNA sequences were shown in Supplementary Table 1.

### RT-qPCR analysis

Total RNA was extracted using Trizol reagent (Invitrogen), and the RNA was reverse-transcribed into cDNA according to the manufacturer's instructions of PrimeScript RT Kit (Takara, Japan). RT-qPCR was performed using a fast SYBR Green PCR kit (Applied Biosystems, Foster City, CA, USA) and ABI PRISM 7300 RT-PCR system (Applied Biosystems) on the Synthesis of cDNA, and the reaction was run in triplicate. GAPDH was used as an internal reference. The relative gene expression was calculated by the 2^-ΔΔCt^ method. The primer sequences were shown in Supplementary Table 1.

### Immunohistochemical (IHC) staining

According to previously described protocols, we used forty samples containing both HCC and adjacent nontumor tissues for the immunohistochemical staining with anti-UBE2O antibody (1:200, 15812-1-AP, Proteintech, Wuhan, China) 22. Two pathologists examined the slides in a double-blind fashion. We randomly selected five high-power fields of view for each section. The intensity of staining was scored as 0 (negative), 1 (weak), 2 (moderate), and 3 (strong), and we scored the percentage of UBE2O positive tumor cells as 1 (0-25%), 2 (26-50%), 3 (51-75%), and 4 (75-100%) 19. The IHC score was equal to the intensity score multiplied by the positive rate score.

### Western blotting

The cells were lysed using RIPA buffer (Boster Biological Technology Ltd., Wuhan, China). The protein concentration was determined based on the manufacturer's instructions of the BCA protein assay kit (Boster). Proteins were separated by 10% SDS-PAGE, and the separated proteins were electro-transferred onto a PVDF membrane (Millipore, Bedford, MA, USA). The membrane was probed with diluted primary antibodies followed by overnight incubation at 4 °C. The next day, the membrane was labeled with HRP-bound secondary antibody incubation (1:1000, Beyotime, Shanghai, China) and detected using an ECL system (Millipore). The resulting bands were scanned using the Amersham Imager 680 machine (GE Healthcare Life Sciences, Pittsburgh, PA, USA). The used primary antibodies were listed in Supplementary Table 2.

### Cell proliferation assay

For the cell counting kit-8 (CCK-8) assay, 2×10^4^ transfected cells were resuspended in 1 mL complete DMEM medium and seeded into a 96-well plate (100 μL for each well). 10 μL of CCK-8 solution (Beyotime) was added to each well of the plate and incubated for 4 h. The absorbance at 450 nm was detected using a Multiskan FC microplate reader (Thermo Fisher Scientific). The EdU assay was carried out using the Cell-Light™ EdU Apollo®488 *In Vitro* Imaging Kit (RIBOBIO, Guangzhou, China) following the manufacturer's protocol, as previously described 23.

### Transwell assay

For migration assay, 3×10^4^ cells were resuspended in serum-free DMEM medium and subsequently placed in the upper chamber, while we added DMEM medium containing 10% FBS to the lower chamber. The cells were cultured at 37 °C for 24 h. The total migrated cells to the lower chamber were fixed with 4% paraformaldehyde, stained with 0.1% crystal violet, and observed under the microscope. For invasion assay, the transwell chamber was covered with Matrigel (BD Biosciences, San Jose, CA, USA). Other operations were the same as the migration assay. The number of migrated and invaded cells were counted to evaluate cell migration and invasion abilities.

### Ubiquitination detection

HCC cells were lysed with an IP buffer containing 0.5% NP40. Cell lysates containing equal volumes and equal amounts of proteins were incubated with 4 µg anti-AMPKα2 antibody (18167-1-AP, Proteintech) and 30 µl slurry of Protein G Sepharose (GE Healthcare Life Sciences) for overnight to pull down AMPKα2 and its associated proteins. The beads were precipitated by centrifugation on the following day, and Ub in the precipitates was quantitated by WB using anti-Ub antibody (1:2000, PTM-1107, PTM BIO, Hangzhou, China).

### TCGA data analysis

The expression of UBE2O and its prognostic significance in the HCC cohort from the TCGA database was performed using the gene expression profiling interactive analysis (GEPIA) webserver (http://gepia.cancer-pku.cn/) according to the manufacturer's protocol 24. The correlation between UBE2O level and tumor stage and tumor grade of HCC was analyzed using UALCAN (http://ualcan.path.uab.edu/analysis.html) 25.

### Statistical analysis

The analysis of variance (ANOVA) with Tukey's multiple comparison test, Student's *t*-test, chi-squared test, and Mann-Whitney U-test were carried out using GraphPad Prism version 8 (GraphPad Inc., San Diego, CA, USA). The survival of two HCC subgroups was compared by the Kaplan-Meier method and log-rank test. Results from at least three independent repeats were shown as mean ± S.D. P<0.05 was considered statistically significant.

## Results

### The overexpression of UBE2O is correlated with HCC patients' survivals

**Figure 1:**
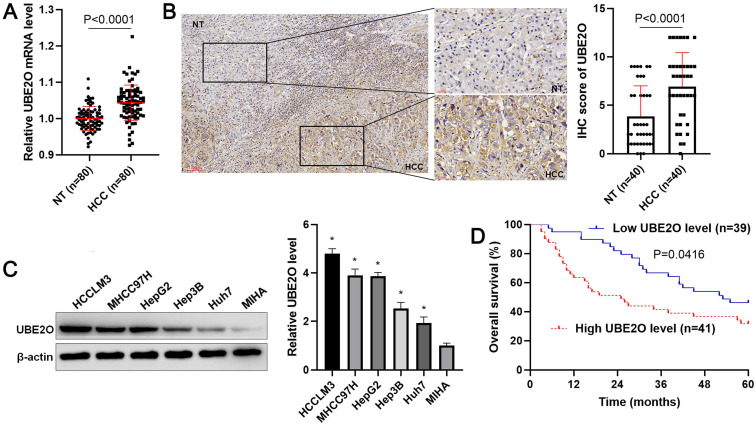
** The expression and survival analysis of UBE2O in HCC. (A)** The levels of UBE2O mRNA were detected in 80 pairs of HCC and nontumor (NT) tissues. **(B)** Left: The presentative result of UBE2O staining in a sample containing both HCC and adjacent NT tissues. Right: The IHC score of UBE2O in HCC (n=40) was remarkably higher than that in adjacent NT tissues (n=40). **(C)** UBE2O expression was assessed in MIHA, HCCLM3, Huh7, HepG2, Hep3B, and MHCC97H cells. n=three independent repeats, *P<0.05. **(D)** We compared the survival of HCC patients with low (n=39) or highly (n=41) expressed UBE2O.

Initially, our RT-qPCR data demonstrated that UBE2O expression was prominently increased in HCC tissue samples compared with adjacent noncancerous tissues (P<0.0001, Figure 1A). Consistent with our data, TCGA data from the GEPIA web server 24 also indicated that UBE2O expression in HCC was markedly higher than that in normal tissues (P<0.05, Supplementary Figure 1A). We used forty samples containing both HCC and adjacent nontumor tissues for the immunohistochemical staining of UBE2O. The IHC score of UBE2O in HCC was remarkably higher than in adjacent nontumor tissues (P<0.0001, Figure 1B). The upregulated expression of UBE2O was verified in HCC cell lines, including HCCLM3, Huh7, Hep3B, HepG2, and MHCC97H compared to MIHA cells (P<0.05, Figure 1C). Table 1 showed that the high level of UBE2O was associated with advanced TNM stage (P=0.004), venous infiltration (P=0.041), and high Edmondson-Steiner grade (P=0.037). The higher expression of UBE2O was detected in advanced and poorly differentiated HCCs, as suggested by TCGA data from the UALCAN website 25 (P<0.05, Supplementary Figure 1B). Next, our follow-up results and TCGA data consistently suggested that the increased level of UBE2O indicated the poor prognosis of HCC (P<0.05, Figure 1D and Supplementary Figure 1C). Accordingly, our data showed UBE2O as a potential predictive marker for HCC prognosis.

### UBE2O facilitates the proliferation and invasion of HCC cells

**Figure 2:**
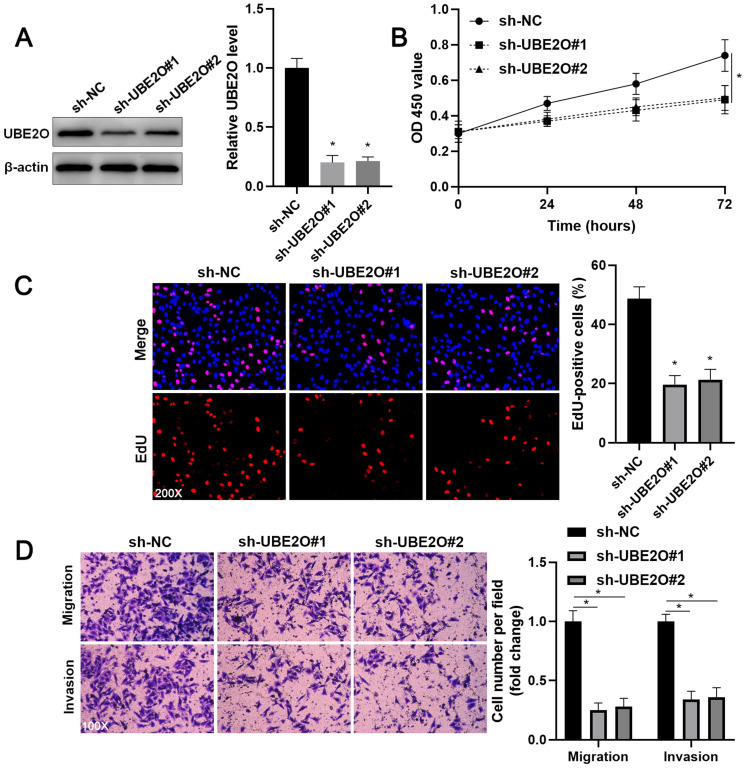
** UBE2O knockdown inhibits HCCLM3 cell proliferation and mobility. (A)** HCCLM3 cells were transfected with nontargeting shRNA (sh-NC) or UBE2O shRNAs (sh-UBE2O#1 and sh-UBE2O#2) and analyzed by immunoblotting for UBE2O expression at 72 h after transfection. **(B)** UBE2O silencing repressed HCCLM3 cell viability, as revealed by the CCK-8 assay. **(C)** EdU assay confirmed that UBE2O knockdown decreased HCCLM3 cell proliferation at 72 h after transfection. **(D)** The number of migrated and invaded HCCLM3 cells was reduced by UBE2O depletion at 72 h after transfection. n=three independent repeats, *P<0.05.

**Figure 3:**
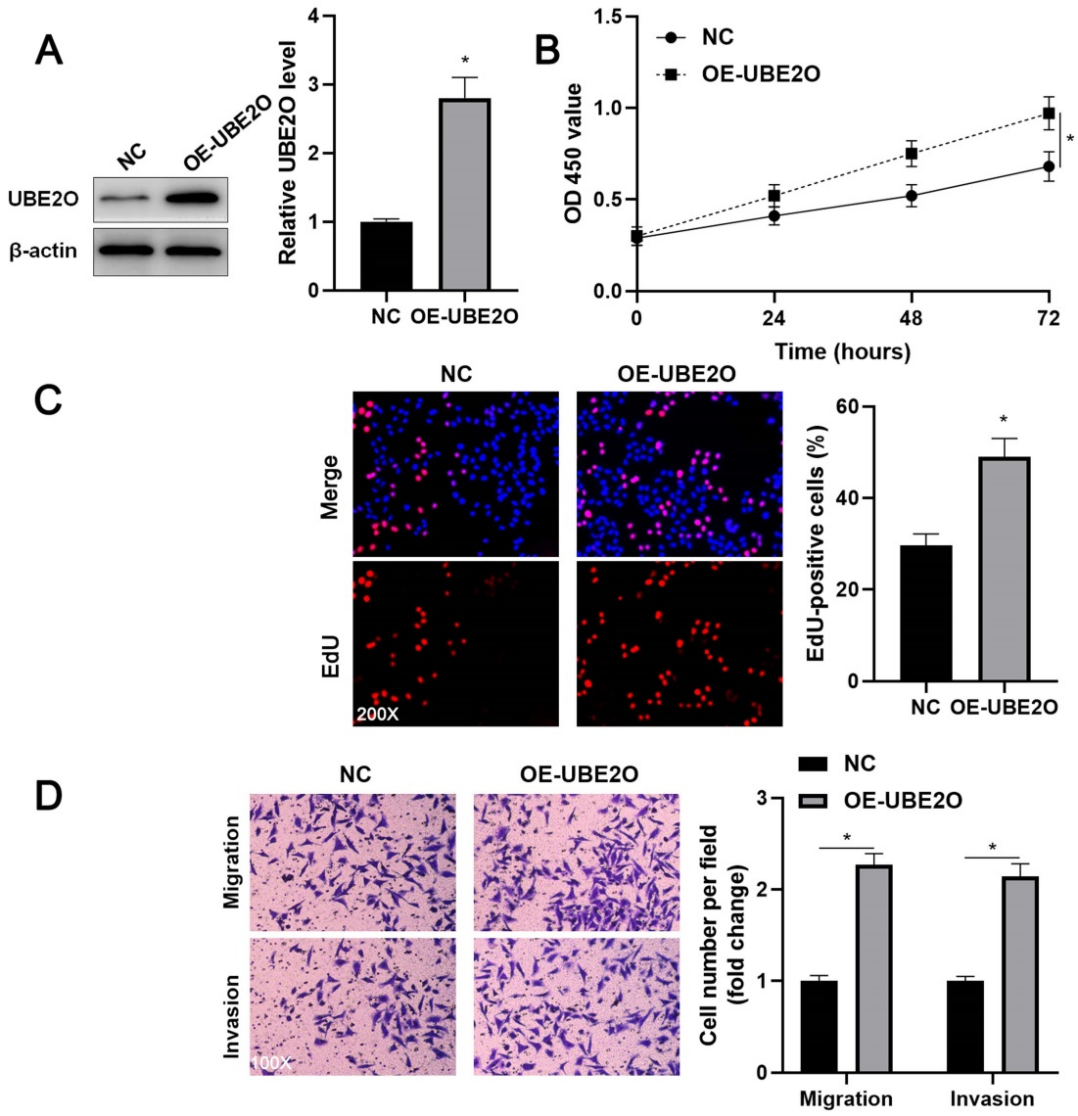
** UBE2O overexpression promotes Huh7 cell proliferation and mobility. (A)** Huh7 cells were transfected with pcDNA3.1 vector carrying UBE2O (OE-UBE2O) or empty vector (NC) and analyzed by immunoblotting for UBE2O expression at 72 h after transfection. **(B)** UBE2O overexpression facilitated Huh7 cell viability, as revealed by the CCK-8 assay. **(C)** EdU assay confirmed that UBE2O upregulation enhanced Huh7 cell proliferation at 72 h after transfection. **(D)** The number of migrated and invaded Huh7 cells was increased by UBE2O overexpression at 72 h after transfection. n=three independent repeats, *P<0.05.

Two independent shRNA constructs downregulated UBE2O expression in HCCLM3 cells (P<0.05, Figure 2A). The viability of HCC cells was significantly repressed by UBE2O knockdown (P<0.05, Figure 2B), as suggested by the CCK-8 assay. UBE2O silencing markedly reduced the percentage of EdU positive HCC cells (P<0.05, Figure 2C) and repressed cell clone formation ability (P<0.05, Supplementary Figure 2). Transwell assays showed that UBE2O depletion remarkably reduced the number of migrated and invaded HCC cells (P<0.05, Figure 2D). Next, UBE2O was ectopically expressed in Huh7 cells (P<0.05, Figure 3A). The functional assays revealed that UBE2O overexpression facilitated the migration, invasion, and proliferation of HCC cells (P<0.05, Figure 3B-3D and Supplementary Figure 2). The effects of UBE2O alteration also confirmed in MHCC97H and Hep3B cells, respectively (P<0.05, Supplementary Figure 3). Therefore, these results suggested that UBE2O enhanced the malignant behaviors of HCC cells.

### UBE2O activates the mTOR pathway by reducing AMPKα2 level

**Figure 4:**
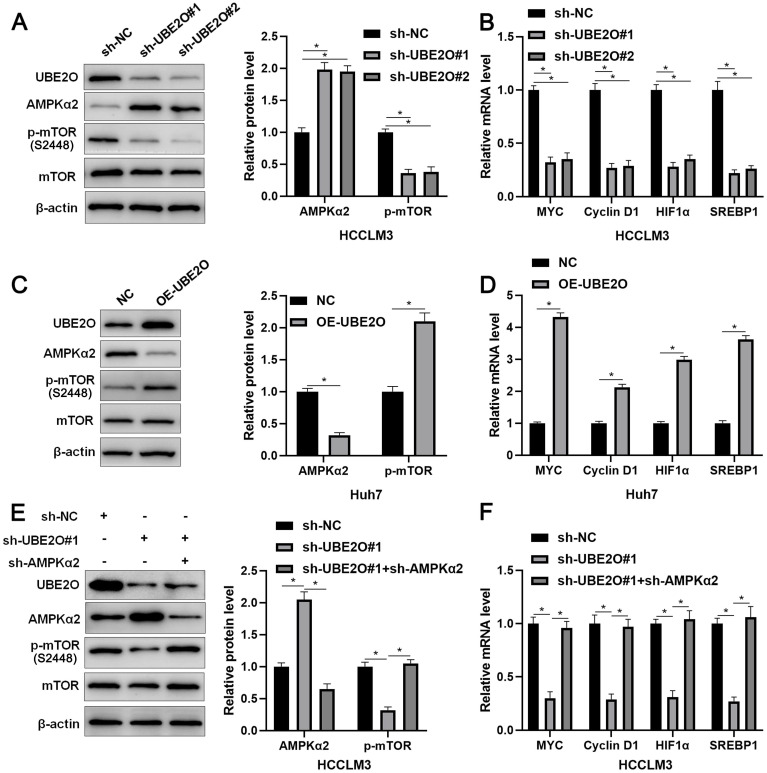
** UBE2O regulates the AMPKα2/mTOR pathway in HCC. (A)** HCCLM3 cells were transfected with sh-NC or UBE2O shRNAs (sh-UBE2O#1 and sh-UBE2O#2) and analyzed by immunoblotting for UBE2O, AMPKα2, p-mTOR, and mTOR levels at 72 h after transfection. **(B)** UBE2O knockdown reduced MYC, Cyclin D1, HIF1α, and SREBP1 mRNA levels in HCCLM3 cells at 72 h after transfection. **(C)** Huh7 cells were transfected with OE-UBE2O or NC and detected by immunoblotting for UBE2O, AMPKα2, p-mTOR, and mTOR levels at 72 h after transfection. **(D)** The ectopic expression of UBE2O upregulated MYC, Cyclin D1, HIF1α, and SREBP1 mRNA levels in Huh7 cells at 72 h after transfection. **(E and F)** Immunoblotting and RT-qPCR analysis found that the AMPKα2 knockdown reversed UBE2O silencing-induced the mTOR pathway inactivation in HCCLM3 cells at 72 h after transfection. n=three independent repeats, *P<0.05.

AMPKα2, a classical substrate of UBE2O, is downregulated in HCC and participates in tumor progression by regulating the mTOR pathway 26, 27. Thus, we intended to confirm whether UBE2O controlled AMPKα2 abundance in HCC. As expected, we found that UBE2O knockdown significantly increased while UBE2O overexpression markedly reduced AMPKα2 protein level (P<0.05, Figure 4A and 4C) without altering AMPKα2 mRNA level in HCC cells (Supplementary Figure 4A). UBE2O knockdown reduced the ubiquitination of AMPKα2 in HCCLM3 cells (Supplementary Figure 4B). UBE2O-induced AMPKα2 downregulation was reversed by a proteasome inhibitor MG132 in Huh7 cells (Supplementary Figure 4C). Moreover, UBE2O positively regulated p-mTOR level but not total mTOR expression in HCC cells (P<0.05, Figure 4A and 4C). The target genes of the mTOR pathway, including p-p70S6K, p-4EBP1, MYC, Cyclin D1, HIF1α, and SREBP1, were also positively regulated by UBE2O in HCC cells (P<0.05, Figure 4B and 4D and Supplementary Figure 5). AMPKα2 knockdown markedly reversed UBE2O silencing-induced the mTOR pathway inactivation in HCCLM3 cells (P<0.05, Figure 4E and 4F). Collectively, UBE2O regulated the AMPKα2/mTOR pathway in HCC cells.

### The mTOR inhibitor blocks the oncogenic role of UBE2O in HCC cells

**Figure 5:**
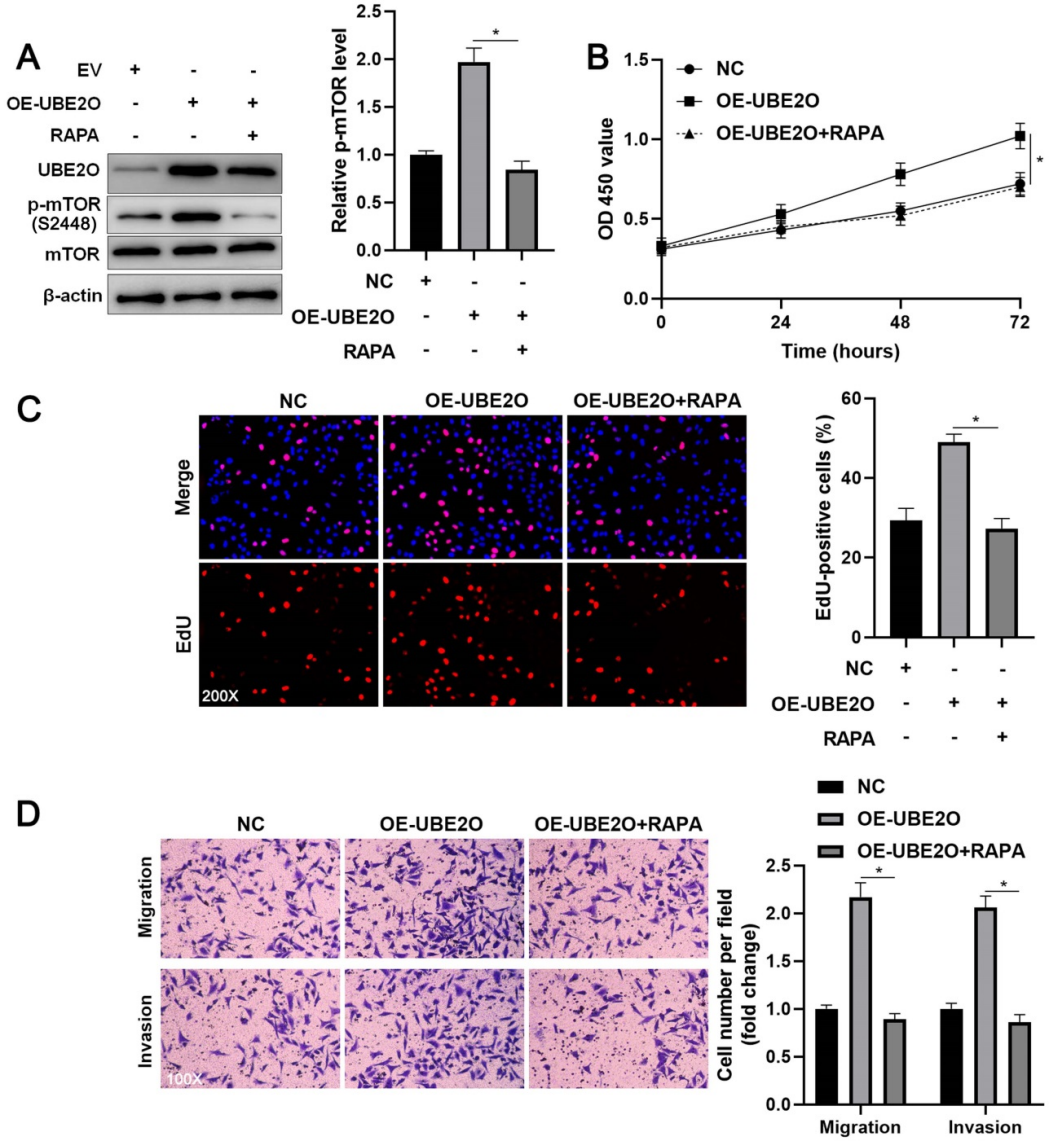
**RAPA treatment reverses the promoting role of UBE2O in HCCLM3 cells. (A)** UBE2O overexpressing HCCLM3 cells were treated with RAPA (10 ng/mL) for 48 h, a specific mTOR inhibitor. UBE2O-induced p-mTOR upregulation was markedly reduced by RAPA treatment. **(B)** MTT, (**C**) EdU, and (**D**) transwell analyses demonstrated that RAPA treatment reversed the tumor-promoting role of UBE2O in HCCLM3 cells. n=three independent repeats, *P<0.05.

To investigate whether UEB2O exerts a tumor-promoting role by activating the mTOR pathway, rapamycin (RAPA), a specific mTOR inhibitor, was used to treat UBE2O overexpressing HCCLM3 cells. As shown in Figure 5A, UBE2O-induced p-mTOR upregulation was prominently reduced by RAPA treatment (P<0.05). RAPA treatment significantly repressed cell proliferation in UBE2O overexpressing HCCLM3 cells, as indicated by CCK-8 and EdU assays (P<0.05, Figure 5B and 5C). Furthermore, RAPA treatment remarkably abrogated the promoting role of UBE2O in HCCLM3 cell mobility (P<0.05, Figure 5D). Thus, UBE2O activated the mTOR pathway to promote HCC progression.

## Discussion

The dysregulation of UBE2O and its clinical significance has been previously reported in several types of human cancers. UBE2O is highly expressed in lung cancer tissues, and its overexpression predicts poor prognosis 19. The overexpression of UBE2O is confirmed in breast cancer tissues, and UBE2O expression is higher in patients with a poor prognosis and an increased risk of metastasis 18. According to TCGA and GEO databases, UBE2O upregulation that is frequently detected in breast, bladder, liver, lung, esophageal, and head and neck cancer predicts poor clinical outcomes in breast, lung, and gastric cancer 16. In this study, we verified that UBE2O expression was prominently elevated in HCC. Then, we revealed that the increased level of UBE2O was closely correlated with advanced tumor stage, venous infiltration, high tumor grade, and poor prognosis of HCC. Thus, UBE2O might be a promising novel prognostic biomarker of HCC. However, the sample size of this study is not large enough, and there is no validation cohort. We will expand the sample size and further clarify the clinical significance of UBE2O in the validation cohort. Previous studies show that epigenetic and transcriptional regulations are implicated in the dysregulation of UBE2O in human cancers 18, 28. Thus, it will be necessary to disclose further the mechanism underlying the upregulation of UBE2O in HCC.

Functional experiments confirmed that UBE2O acted as an oncogene in HCC and facilitated the proliferation and mobility of tumor cells. The UBE2O/AMPKα2 axis has been demonstrated in skeletal muscle and breast cancer 16, 18, 20. UBE2O targets AMPKα2 for ubiquitination and degradation, thereby activating the mTOR pathway in breast cancer 16, 18. Here, we found that UBE2O also inversely regulated the AMPKα2 level in HCC cells without affecting the AMPKα2 mRNA level. We further confirmed the impact of UBE2O in the ubiquitination and degradation of AMPKα2 in HCC. Several other proteins, such as Mxi1, BAP1, c-Maf, and SMAD6, are recognized as substrates of UBE2O 15, 17, 19, 29. Thus, it is worth checking the levels of these proteins after modulating UBE2O expression and discover novel targets for UBE2O in HCC cells. Next, we found that UBE2O positively modulated the mTOR pathway in HCC cells. Moreover, AMPKα2 knockdown markedly reversed UBE2O silencing-induced the inactivation of the mTOR pathway. AMPKα2 is previously reported to be low expressed in HCC and contributes to hepatocarcinogenesis by acetylating and stabilizing p53 26. AMPKα2 is also implicated in glycolic metabolism and chemoresistance of HCC 30. Many studies have demonstrated that the mTOR pathway is one of the essential oncogenic pathways negatively regulated by AMPK in HCC 31-33. mTOR forms a catalytic subunit of two distinct protein complexes, mTOR complex 1 (mTORC1) and mTORC2 34. Activation of mTORC1 most prominently results in phosphorylation of two downstream targets, p70S6K and 4EBP1, promoting protein synthesis 35. 4EBP1 and p70S6K axes play critical and distinct roles in hepatocarcinogenesis 36. The target genes of the mTOR pathway, such as MYC, Cyclin D1, HIF1α, and SREBP1, participate in many malignant behaviors of HCC cells 37-39. Recent studies report that glutamine synthetase (GS), a transcriptional target of β-catenin, functions as a driver in HCC development by promoting p-mTOR (S2448) expression 40-42. RAPA, a specific mTOR inhibitor, prominently abolished UBE2O-induced the mTOR pathway activation and HCC cell proliferation and mobility. Collectively, UBE2O exerted the pro-HCC effects by activating the AMPKα2/mTOR pathway. In this study, we did not provide animal experimental data to support the functional identification and molecular mechanism analysis of UBE2O in HCC. Therefore, it is necessary to perform the animal experiments in further study.

To conclude, we demonstrated the upregulation of UBE2O in HCC and verified their potential in predicting poor prognosis. UBE2O promoted the migration, invasion, and proliferation of tumor cells by activating the AMPKα2/mTOR pathway in HCC. UBE2O might be promising prognostic biomarkers and new therapeutic targets of HCC.

## Supplementary Material

Supplementary figures and tables.

## Figures and Tables

**Table 1:** Clinicopathological correlation of UBE2O expression in human hepatocellular carcinoma

Characteristics	n=80	UBE2O		*P*
		Low expression (n=39)	High expression (n=41)	
**Age (years)**				0.352
<50	35	15	20	
≥50	45	24	21	
**Sex**				0.481
Male	63	32	31	
Female	17	7	10	
**HBV infection**				0.527
No	28	15	13	
Yes	52	24	28	
**Serum AFP level (ng/mL)**				0.385
<20	27	15	12	
≥20	53	24	29	
**Tumor size (cm)**				0.267
<5	26	15	11	
≥5	54	24	30	
**No. of tumor nodules**				0.185
1	65	34	31	
≥2	15	5	10	
**Cirrhosis**				0.185
No	35	20	15	
Yes	45	19	26	
**Venous infiltration**				0.041*
No	44	26	18	
Yes	36	13	23	
**Edmondson-Steiner grade**				0.037*
I+II	57	32	25	
III+IV	23	7	16	
**TNM stage**				0.004*
I+II	63	36	27	
III+IV	17	3	14	

HBV, hepatitis B virus; AFP, alpha-fetoprotein; TNM, tumor-node-metastasis. The “low” or “high” expression of UBE2O level was defined according to the cut-off value, which was defined as the median value of the cohort of patients tested. ^*^Statistically significant.

## Abbreviations

HCC: hepatocellular carcinoma
TRAF7: tumor necrosis factor receptor-associated factor 7
KLF4: Krüppel-like factor 4
FBXW7: F-box and WD repeat domain containing 7
YAP: Yes-associated protein
UBE2S: ubiquitin-conjugating enzyme E2 S
TRIM28: tripartite motif-containing 28
GSK3β: glycogen synthase kinase 3 beta
BAP1: BRCA1-associated protein 1
MLL: mixed-lineage leukemia
AMPKα2: AMP-activated protein kinase α2
mTOR: mechanistic target of rapamycin kinase
