# Supplementary material for: UBE2O promotes hepatocellular carcinoma cell proliferation and invasion by regulating the AMPKα2/mTOR pathway
Source: Int J Med Sci. 2021 Oct 11;18(16):3749–58. doi: 10.7150/ijms.63220 (PMC8579295; doi:10.7150/ijms.63220)
Supplement: Supplementary file 1 — Supplementary figures and tables. [file ijmsv18p3749s1.pdf]

Supplementary Table 1 The primer and shRNA sequences used in this study

Gene	Primer	Sequence (5′-3′)
UBE2O	forward	GAATCCAAAACCAAGAGCGAAG
	reverse	TCATCTCTGCCTTCTTTTAGCA
MYC	forward	TGGAACTTACAACACCCG
	reverse	CCTCGTCGCAGTAGAAAT
Cyclin D1	forward	CTGACACCAATCTCCTCAACG
	reverse	CTCACAGACCTCCAGCATCCA
HIF1α	forward	TTGCTGAAGACACAGAAGCGAAGA
	reverse	TTGCTGAAGACACAGAAGCGAAGA
SREBP1	forward	CTGGTCTACCATAAGCTGCAC
	reverse	GACTGGTCTTCACTCTCAATG
GAPDH	forward	GGAGCGAGATCCCTCCAAAAT
	reverse	GGCTGTTGTCATACTTCTCATGG
**shRNA**		**Sequence (5′-3′)**
sh-UBE2O#1		CCGGCGATGATTCCTATGGCTTCTACTCGAGTAG AAGCCATAGGAATCATCGTTTTT
sh-UBE2O#2		CCGGGACATCAAGAAGCTACAGGAACTCGAGT TCCTGTAGCTTCTTGATGTCTTTTT
sh-AMPKα2		CCGGCGCAGTTTAGATGTTGTTGGACTCGAGTC CAACAACATCTAAACTGCGTTTTT

Supplementary Table 2 The antibodies used for WB in this study

Gene	Dilution	Catalog number	Company
UBE2O	1:1000	15812-1-AP	Proteintech
AMPKα2	1:1000	18167-1-AP	Proteintech
p-mTOR (Ser2448)	1:2000	67778-1-Ig	Proteintech
mTOR	1:5000	66888-1-Ig	Proteintech
MYC	1:1000	10828-1-AP	Proteintech
HIF1α	1:1000	NB100-110	Novus Biologicals
Cyclin D1	1:5000	60186-1-Ig	Proteintech
SREBP1	1:1000	14088-1-AP	Proteintech
p-p70S6K (Thr389)	1:1000	#9234	Cell Signaling Technology
p70S6K	1:1000	#9202	Cell Signaling Technology
p-4EBP1 (Thr37/46)	1:1000	#2855	Cell Signaling Technology
4EBP1	1:1000	#9644	Cell Signaling Technology
β-actin	1:5000	66009-1-Ig	Proteintech

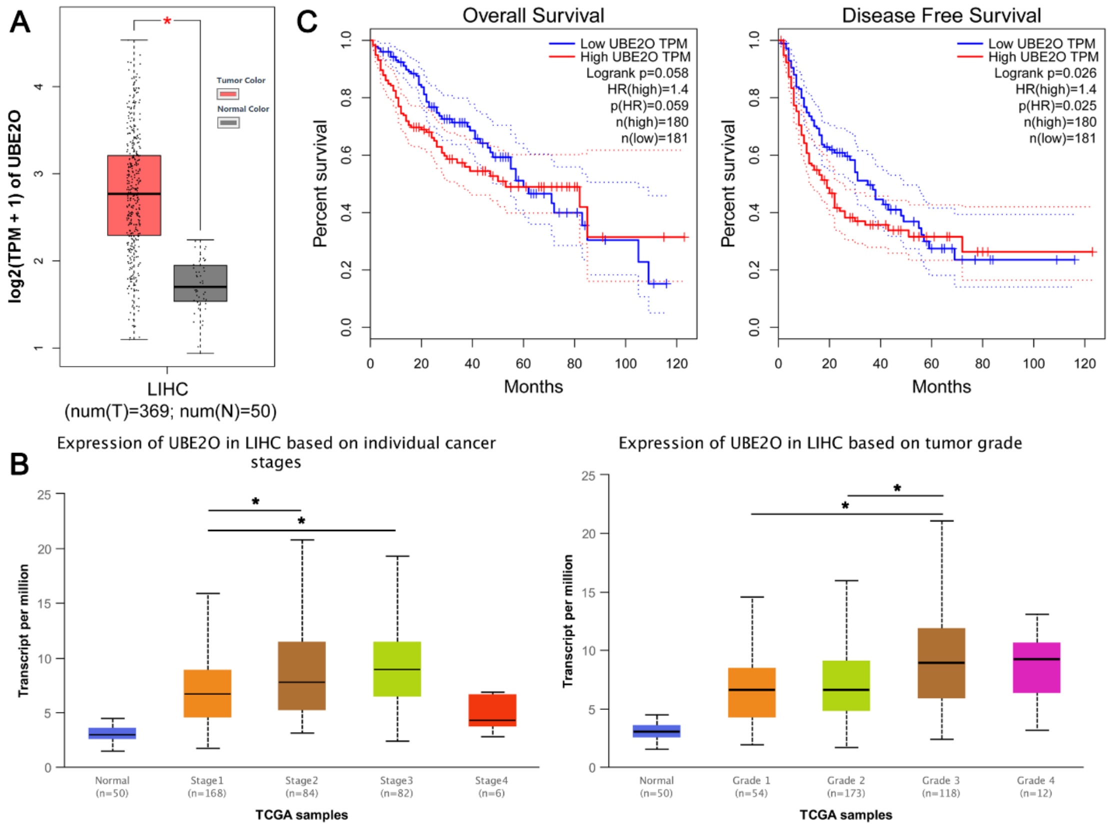

Supplementary Figure 1 The clinical significance of UBE2O upregulation in HCC from TCGA data. (A) The expression of UBE2O were compared between HCC and normal tissues. (B) The correlation between UBE2O level and tumor stage and tumor grade of HCC, respectively. (C) The high expression of UBE2O indicated the poor prognosis of HCC. *P<0.05.

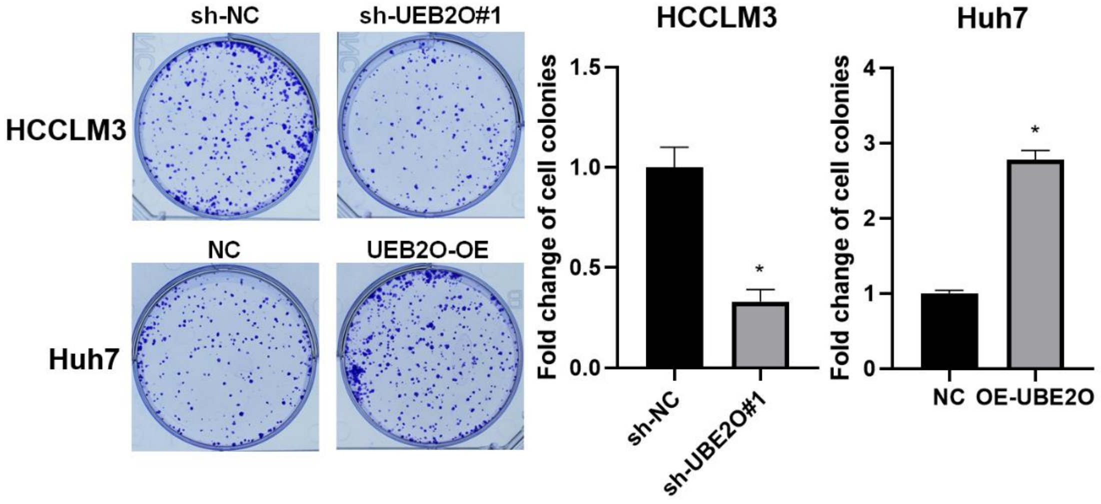

Supplementary Figure 2 UBE2O promotes the colony formation ability of HCC cells. n=three independent repeats, *P<0.05.

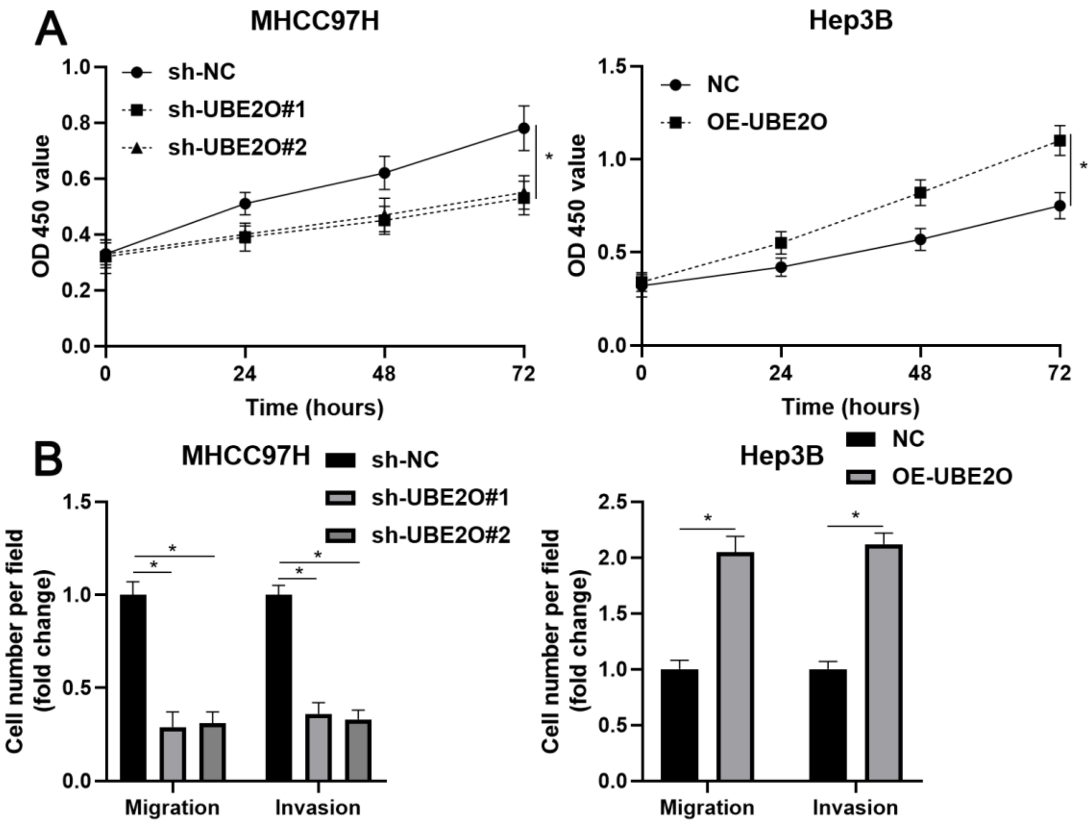

Supplementary Figure 3 UBE2O promotes the proliferation and mobility of HCC cells. (A) UBE2O knockdown repressed the proliferation of MHCC97H cells and UBE2O overexpression facilitated Hep3B cell proliferation. (B) UBE2O silencing inhibited but UBE2O overexpression enhanced the migration and invasion potentials of HCC cells. n=three independent repeats, *P<0.05.

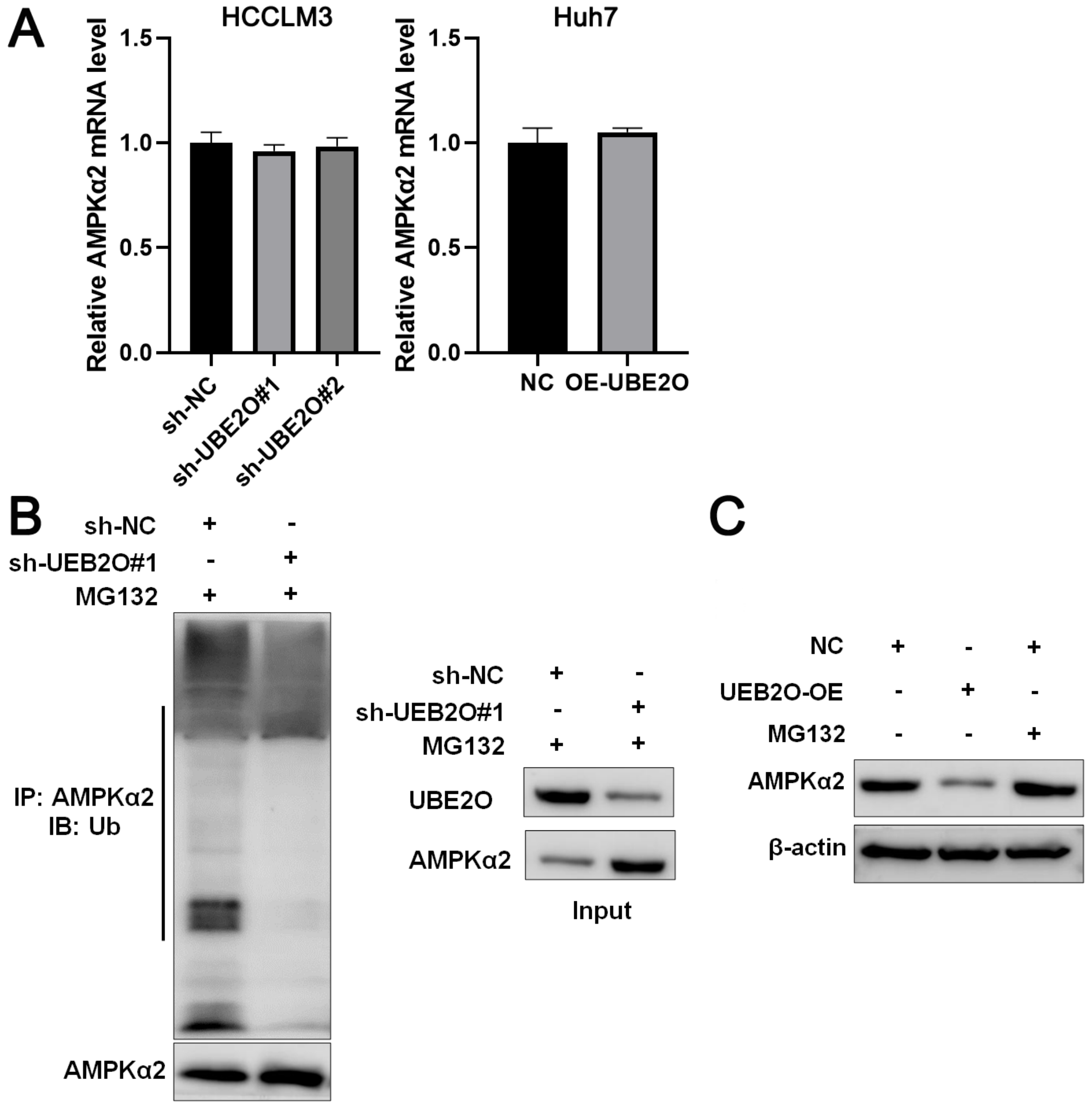

Supplementary Figure 4 UBE2O affects the ubiquitination and degradation of AMPKα2 in HCC cells. (A) The AMPKα2 mRNA level was not affected by UBE2O alteration in HCC cells. (B) UBE2O knockdown reduced the ubiquitination of AMPKα2 in HCCLM3 cells. (C) UBE2O-induced AMPKα2 downregulation was abolished by a proteasome inhibitor MG132 in Huh7 cells.

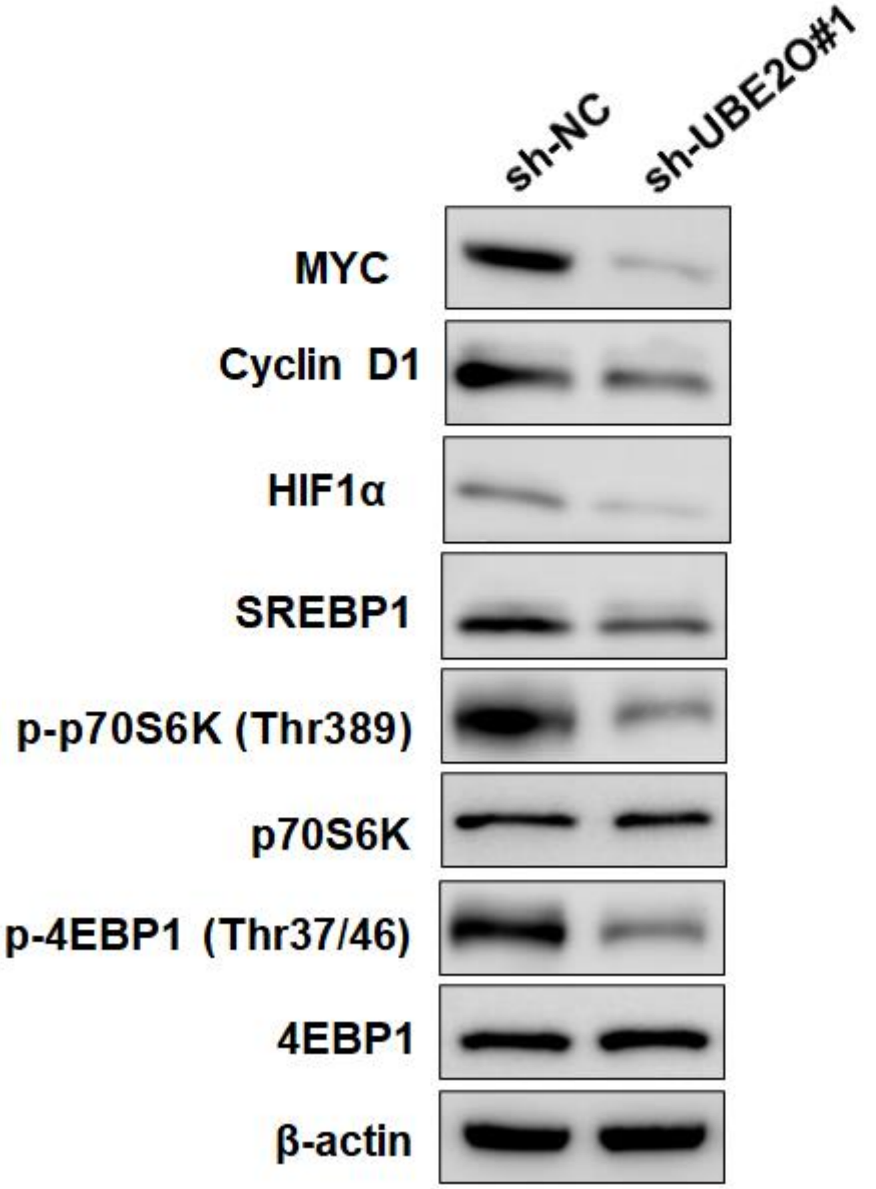

Supplementary Figure 5 UBE2O knockdown reduced the levels of MYC, Cyclin D1, HIF1α, SREBP1 protein in HCCLM3 cells.
